# A comparison of the costs and patterns of expenditure for care for severe mental illness in five countries with different levels of economic development

**DOI:** 10.1017/S2045796025100140

**Published:** 2025-07-16

**Authors:** A-La Park, Oliver Jez, Reinhold Kilian, Ashleigh Charles, Jasmine Kalha, Palak Korde, Max Lachmann, Candelaria Mahlke, Galia Moran, Juliet Nakku, F. Ngakongwa, Jackline Niwemuhwezi, Rebecca Nixdorf, Grace Ryan, Donat Shamba, Mike Slade, T. Waldmann

**Affiliations:** 1 Care Policy and Evaluation Centre, Department of Health Policy, London School of Economics and Political Science, London, UK; 2 Department of Psychiatry and Psychotherapy II, University of Ulm and BKH Günzburg, Günzburg, Germany; 3 School of Health Sciences, Institute of Mental Health, University of Nottingham, Nottingham, UK; 4 Centre for Mental Health Law and Policy, Indian Law Society, Pune, India; 5 Department of Social Work, Ben Gurion University of the Negev, Be’er Sheva, Israel; 6 Department of Psychiatry, University Medical Center Hamburg-Eppendorf, Hamburg, Germany; 7 Butabika National Referral Hospital, Kampala, Uganda; 8 Ifakara Health Institute, Dar es Salaam, Tanzania; 9 Centre for Global Mental Health, Department of Population Health, London School of Hygiene and Tropical Medicine, London School of Hygiene and Tropical Medicine, London, UK; 10 Faculty of Nursing and Health Sciences, Health and Community Participation Division, Nord University, Bodø, Norway

**Keywords:** economic issues, health economics, health service research, mental health

## Abstract

**Aims:**

The aim of the article is to undertake the first economic analysis exploring the costs of illness (COIs) and factors affecting COIs in people living with mental disorders using individual patient-level data across five countries with different national income levels. This is done by investigating diagnosis-related and sociodemographic factors for country-specific medical and psychosocial service use in these high, lower-middle and low-income countries.

**Methods:**

Using data from the Using Peer Support In Developing Empowering Mental Health Services (UPSIDES) study, a pragmatic randomized controlled trial, costs for medical and psychosocial services have been estimated over 6 months in 615 people with severe mental illness from Germany (*n* = 171), Uganda (*n* = 138), Tanzania (*n* = 110), India (*n* = 93) and Israel (*n* = 103). The primary economic analysis included (1) total COI expressed in 2021 international dollars and (2) proportional cost-type expenditures. Generalized linear regression models were also used to estimate the impact of psychiatric diagnosis, social disability, age and gender on the total COI.

**Results:**

Of the 615 participants (mean [SD] age 38.3 [11.2] years; 335 [54.5%] women), the total 6-month COI ranged from $311.48 [±547.47] in Tanzania to $10,493.19 [±13324.10] in Germany. High-income Germany and low-income Uganda both concentrated >70% of COIs on inpatient care. High-income Israel had the most balanced COI, with the lowest mean share (15.40%) on inpatient care, compared with community (35.12%) and primary care (33.01%). Female gender was associated with lower COI (*e*^b^ = 0.215; *p* = 0.000) in Tanzania, while in India diagnosis of depression was associated with lower costs than schizophrenia (*e*^b^ = 0.363; *p* = 0.017). Health of the Nation Outcome Scale scores (social disability) were not significantly associated with COIs in any country. In Tanzania, the total mean COI increased by 3.6% for every additional year of age. Compared to Germany, mean COIs were significantly lower by 90%, 99% and 86% in Uganda, Tanzania and India, respectively, and by 50% in Israel, although this difference was not significant.

**Conclusions:**

National income is correlated with the total COI in people living with mental disorders but is a poor predictor of the sector-specific distribution of these expenditures.

## Introduction

Mental disorders are a major contributor to the global burden of disease (GBD) (GBD 2019 Mental Disorders Collaborators, 2022). They also affect most dimensions of human life, having a high level of social stigmatization (Patel *et al.*, 2018). The case for more investment in mental health has long been made, and mental healthcare expenditure has increased significantly, at least in many wealthy countries (Christensen *et al.*, 2020). The World Health Organization (WHO) has also argued that the importance of investing more resources into mental healthis also about additional impacts on physical health; for example, people living with major depression or schizophrenia have a 40%–60% greater chance of premature mortality compared to people without mental disorders (World Health Organization, 2021a).

The attention given to mental health in high-income countries may, in part, reflect data indicating that the prevalence and number of disability-adjusted life years (DALYs) attributable to mental disorders are much higher in high-income countries, i.e. in countries classified by the World Bank in 2024–2025 as having a gross national income (GNI) per capita of more than $14,005, compared with lower-middle income countries with GNI per capita between $1,146 and $4,515 or low-income countries with a GNI per capita of less than $1,146 per capita (Metreau *et al.*, 2024).

One caveat, however, is that the GBD burden in low- and lower-middle-income countries in sub-Saharan Africa varies substantially, so there is considerable uncertainty around differences in GBD between countries because of limited epidemiological data in Africa (Omigbodun *et al.*, 2023). Countries in the lowest income categories are less likely to ensure a minimal level of medical treatment or psychosocial care for those with the most severe mental illnesses (OECD, 2021; World Health Organization, 2021b). The mean number of psychiatric hospital beds per 100,000 population in 2017 was only 1.9 in low-income countries, in contrast to 28.6 in high-income countries (World Health Organization, 2021b).

Given that international action plans recommend a shift in the care balance from hospital to community-based care (World Health Organization, 2021a), as well as integrating mental health into primary care in low- and middle-income countries (Thornicroft and Tansella, 2013), these disparities are even more stark. There are 30 community mental health outpatient visits per 100,000 population in low-income countries, in contrast to about 3,000 in high-income countries (World Health Organization, 2021b). In addition to disparities between high- and low-income countries, there are also large differences between countries within the same income categories in terms of performance against mental healthcare targets, as defined by the WHO Comprehensive Mental Health Action Plan and OECD mental health benchmark indicators (OECD, 2021; World Health Organization, 2021b).

To date, most comparisons on mental health service use have been based on aggregate-level top-down data or expert surveys (Arias *et al.*, 2022; Rajkumar, 2022). Individual patient-level data are rarely available (Addo *et al.*, 2018). These data tend to give the overall impression of a lack of mental healthcare resources and infrastructure. However, they do not reveal how people with mental health problems make use of available services or the extent to which the lack of professional mental healthcare can be compensated by social support or other types of informal healthcare, such as traditional healers (Mokgobi, 2014).

Our study aims to compare total costs of illness (COIs) in people living with mental disorders in a standardized way across five countries – covering three of the four categories of the World Bank income scale: Germany and Israel (high income), India and Tanzania (lower middle income) and Uganda (low income). The countries provide a spectrum of policy and practice contexts. The two high-income countries are examples of different approaches to the management of mental disorders. Reform in Israel in 2015 moved responsibility for mental health from the Ministry of Health to four social health insurers with the aim of integrating mental health into general health services with more focus on community-based care (Gal *et al.*, 2021). In contrast, the German system has not experienced this transformation and has continued to operate a model dominated by psychiatric inpatient care and less integration with community services (Wiegand *et al.*, 2025). Indian national mental health policy, coupled with reforms to expand access to publicly funded healthcare, has put a focus on community mental health services (Mahapatra and Seshadri, 2024). Uganda has had a national mental health policy for almost 30 years, which also commits to more community-focused care (Atewologun *et al.*, 2025), while Tanzania represents a country with little history of mental health policy development (Atewologun *et al.*, 2025).

Our study explores whether total COIs are associated with diagnosis, socio-demographic characteristics and Health of the Nation Outcome Scale (HoNOS) scores. It looks at differences in patterns in service utilization, using bottom-up costing methods (Knapp and Beecham, 1990), with individual health service user data. Results are primarily expected to provide insights into the effects of economic disparities on mental health service use and secondarily to have implications for improvement strategies.

## Methods

Contextual information on country characteristics was taken from published literature, including gross domestic product (GDP) per capita (World Bank, 2020), total governmental health expenditure as a percentage of GDP and proportion of expenditure allocated to mental health (GBD 2019 Mental Disorders Collaborators, 2022). Funding structures and mental health service capacity were taken from the WHO Mental Health Atlas’ 2020 (Germany, Tanzania and Uganda) (World Health Organization, 2021b) and 2017 (India and Israel) (Jaeschke *et al.*, 2021).

The investigation makes use of baseline data from the ‘Using Peer Support In Developing Empowering Mental Health Services (UPSIDES)’ study (Moran *et al.*, 2020). UPSIDES is an international multisite trial, assessing the effectiveness of implementing peer support for people with severe mental illness in these five countries.

### Study sample

Participants aged 18–60 years were recruited at psychiatric treatment facilities. Detailed information on study sites and recruitment strategies is found in the study protocol; they included multiple strategies, including ‘outpatient/community mental health services, patient/carer organisations, local newspapers, social media, community leaders, and word of mouth’ (Moran *et al.*, 2020).

Participants needed a mental disorder diagnosis according to case notes, staff communication or self-labelling for at least 24 months, as well as a severity-of-illness threshold of 5 points or more assessed using the Threshold Assessment Grid, with illness duration (2 years and over) (Slade *et al*., 2000). Additionally, participants needed to have the capacity to provide written informed consent in their local language. Exclusion criteria included learning disability, dementia, substance disorder or organic brain disorder diagnoses. Social disability was measured using the HoNOS (score ranges: 0–48, higher scores indicating greater severity) (Wing *et al.*, 1998, 2000).

### Assessment of healthcare use

Use of medical and psychosocial services and support was comprehensively assessed using the Client Sociodemographic and Service Receipt Inventory (CSSRI) (Chisholm *et al.*, 2000), adapted for use in participating countries as the CSSRI-UPSIDES (Charles *et al.*, 2022). For adaptation purposes, mental healthcare experts from participating study sites were consulted to check service categories of the original instrument for applicability and to add and define country or site-specific services or support categories not already included (Moran *et al.*, 2020).

The main categories of CSSRI-UPSIDES are all inpatient and outpatient health services, community mental health services, primary healthcare, justice system services, medication and out-of-pocket payments. Inpatient services include all types of inpatient hospital treatment; outpatient services include all types of outpatient treatment provided by hospitals or by office-based physicians; and community mental health services include psychosocial services provided in the community, such as in day centres, occupational rehabilitation and housing support. Primary healthcare services include all medical services provided in the community free of charge, such as family doctors, community health centres, community nurses and midwives. Justice includes costs for police contacts, detention, time in police custody, imprisonment and trials. Costs for medication cover those taken outside of hospital settings.

### Collection of unit cost information

Country-specific costs for service units were obtained from publicly available sources and consultation with mental health service experts at study sites (see Supplementary eTables 1–5, Supplement 1, for country values). For potential reliability issues, unit costs were triangulated using published information, expert communications and communications with local facility staff members. Due to the unavailability of reliable country-specific cost information for all countries, drug costs were calculated on the basis of the British National Health Service Drug Tariff (National Health Service Business Service Authority (NHSBSA), 2022).

### Estimation of the total cost of illness

Reported service units were multiplied by unit costs in the local currency. Costs for outpatient care over 3 months and costs for medical drugs for 1 month were extrapolated to estimate costs for a 6-month period. To allow comparison, all country-specific costs were converted to one currency (2021 price years), expressed as international dollars (Int$), using purchasing power parity (PPP) (International Monetary Fund, 2022). All relevant elements of the Consolidated Health Economic Evaluation Reporting Standards 2022 (CHEERS 2022) Statement (Husereau *et al.*, 2022) were followed (Supplement 2).

### Statistical analysis

Generalized linear models (GLMs) with log link and gamma distribution of errors were applied to account for skewed distribution of healthcare cost data (Kilian *et al.*, 2002; Mccullagh, 1989). Initially, country-specific models were estimated with gender, age, psychiatric diagnosis and HoNOS total score as independent variables. Subsequently, an overall model was computed including dummy variables for country using Germany as a reference category. To account for country-specific differences, multiplicative interaction terms between country × gender, country × age, country × diagnosis and country × HoNOS were included. To facilitate interpretation, regression coefficients were reported as exponentiated betas. Since the GLM provides no *R*^2^ statistic, we computed *R*^2^ from the correlation between raw costs (*y*) and costs predicted by the regression equation 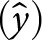 as 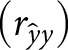
^2^. Diagnostic residual plots were produced to test model requirements. Statistical analyses were conducted in Stata 17 (StataCorp, 2021).

The study was approved by the Ethics Commission of Ulm University, Germany (ref. 254/19), Local Psychological Ethics Commission, Center for Psychological Medicine, Hamburg, Germany (ref. LPEK-0095), Uganda National Council for Science and Technology (ref. SS 4990), National Institute for Medical Research, Das es Salaam, and Ministry of Health, Community Development, Gender, Elderly & Children, Dodoma, Tanzania (ref. NIMR/HQ/R.8a/Vol.IX/3328), the Human Subjects Research Committee of Ben-Gurion University, Israel (ref. 1878-1) and the Indian Law Society (ref. LIS/37/2018).

## Results

Table 1 provides information on country characteristics. GDP per capita ranged from $2,275 in Uganda to $54,551 in Germany, a 24-fold difference. GDP percentage spent on overall healthcare varied from 2.96% in India to 13% in Germany. Mental health expenditure as a percentage of the total health expenditure ranged from 1.3% in India to 13.1% in Germany. Mental healthcare in Germany and Israel was mainly funded by statutory health insurance, with 90% and 95% coverage, respectively. That said, 3.4% overall spending on mental health in Israel as a proportion of total governmental health expenditure is similar to the three other countries in our analysis. However, India, Tanzania and Uganda relied predominantly on out-of-pocket payments and private health insurance schemes, while in Israel, mental health is covered by the statutory health system.

**Table 1.** Comparisons of healthcare systems in five countries

	Germany	Israel	India	Tanzania	Uganda
World Bank income level	High	High	Lower-middle	Lower-middle	Low
GDP per capita (Int$)	$54,551	$41,271	$6,971.7	$2,772.9	$2,275
% GDP spent on healthcare	13%	7.5%	2.96%	3.75%	3.96%
% healthcare on mental health	13.1%	3.4%	1.3%	4%	2.9%
Prevalence of depression/100,000 pop.	2,392.3	3,079.6	2,547.8	3,295.7	5,337.6
Prevalence of schizophrenia/100,000 pop.	262.7	299.1	285.2	313.3	207.9
DALYs/100,000 pop. due to mental disorders	2,375.2	1,906.2	1,561.6	1,721.6	2,153.3
Psychiatrists/100,000 pop.	14.22	9.87	0.29	0.07	0.09
Psychologists/100,000 pop.	55.08	88.09	0.07	0.03	0.10
Psychiatric hospital beds/100,000 pop.	159.87	40	2.01	2.34	3.68
Main method of financing	Statutory health insurance	Statutory health insurance	Mainly out-of-pocket payment	Out-of-pocket or private insurance	Out-of-pocket or private insurance

*Source*: Mental Health Atlas 2020 country profiles (2017 profile for India and Israel), Global Burden of Disease Study 2019.

While prevalence rates for schizophrenia were similar among the five countries, the prevalence of depression per 100,000 population was highest at 5,337.6 in Uganda and lowest at 2,392.3 in Germany (GBD 2019 Mental Disorders Collaborators, 2022). Rates for DALYs per 100,000 population varied between 1,561.6 in India and 2,153.3 in Uganda (GBD 2019 Mental Disorders Collaborators, 2022).

Regarding workforce, the number of psychiatrists ranged from 0.06 per 100,000 population in Tanzania to 13 in Germany (World Health Organization, 2021b). Israel had the highest number of psychologists per 100,000 population (88.09), while this ranged from 0.01 to 0.08 in lower-middle and low-income countries. Germany had the highest number of psychiatric hospital beds (150 per 100,000), followed by Israel (40), whereas very low rates were reported in the other countries (1.34–2.45).

Baseline characteristics of all 615 participants are reported in Table 2. The mean age was 38.3 (SD 11.2 years), and the study was broadly balanced; 54.5% were women. The mean HONOS score was 14.8, ranging from 19.6 in Germany to 6.8 in Uganda. In total, 54% had a depression diagnosis.

**Table 2.** Characteristics of study participants

	Total	Germany	India	Israel	Tanzania	Uganda
*N* (%)	615 (100)	171 (27.8)	93 (15.1)	103 (16.8)	110 (17.9)	138 (22.4)
Age (SD)	38.3 (11.2)	39.8 (13.0)	38.7 (9.4)	45.1 (10.5)	34.1 (8.6)	34.5 (9.5)
Women, *N* (%)	335 (54.5)	121 (70.8)	45 (48.4)	52 (50.5)	58 (52.7)	59 (42.8)
HoNOS (SD)	14.8 (8.41)	19.6 (6.6)	18.0 (8.1)	15.6 (7.0)	13.7 (7.5)	6.8 (6.0)
Diagnosis, *n* (%)						
• Schizophrenia	197 (32.0)	23 (13.5)	21 (22.6)	45(43.7)	56 (50.9)	52(37.7)
• Depression	336 (54.6)	90 (52.6)	67 (72.0)	48 (46.6)	46 (41.8)	85 (61.6)
• Other	72 (11.7)	57 (33.3)	5 (5.4)	10 (9.7)	0 (0.0)	0 (0.0)

## Total costs for healthcare in five countries

**Figure 1.**
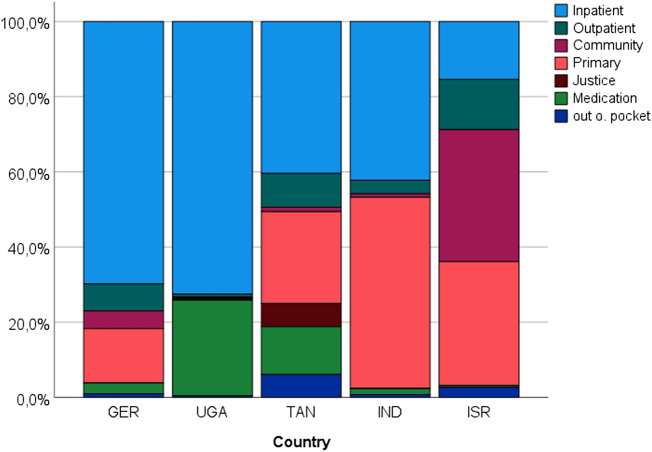
Distribution of total healthcare expenditure (GER = Germany; UGA = Uganda; TAN = Tanzania; IND = India; ISR = Israel).

Table 3 shows total healthcare costs, disaggregated by cost component. Total costs ranged from $311.48 in Tanzania to $10,493.19 in Germany. Comparison of balance between different cost categories in Figure 1 indicates that 69.80% (Germany) and 73.65% (Uganda) of costs were for inpatient care. German respondents also reported 14.48%, 7.17% and 4.73% of expenditures were for primary, outpatient and community care. The almost complete reliance on inpatient care in Uganda, where the only other costs reported were for medications, coupled with the relatively low unit costs for inpatient care, meant a mean of more than 25 nights spent in hospital over the 6-month time period, much higher even than in Germany at almost 20 nights, because of the higher unit costs of care. Mean inpatient stays were approximately 13 nights in India, 3 in Israel and 2 in Tanzania.

**Table 3.** Total healthcare costs in five countries (mean ± SD in Int$)

Services	All (*N* = 615)	Germany (*N* = 171)	Israel (*N* = 173)	Tanzania (*N* = 110)	Uganda (*N* = 138)	India (*N* = 93)
Mean costs ± SD						
Inpatient services	2,515.82	7,323.77	1,071.37	125.84	948.56	427.66
SD	±7,949.17	±13,610.72	±3,871.58	±365.04	±916.44	±1,074.93
Day hospital services	377.82	752.88	928.46	27.99	10.94	36.5
SD	±1,719.63	±3,105.40	±869.63	±83.63	±19.47	±43.95
Psychosocial services	550.02	496.28	2,442.79	3.66	3.55	9.69
SD	±1,602.50	±1,456.92	±2,702.36	±23.69	±19.81	±47.91
Outpatient services	898.59	1,519.13	2,296.1	76.1	1.51	513.81
SD	±1,558.71	±1,891.86	±1,840.79	±257.53	±7.70	±835.34
Police/judicial services	4.84	0	0	19.33	5.21	1.43
SD	±84.39	±0.00	±0.00	±192.24	±46.52	±13.82
Medication	172.4	298.39	30.84	39.56	312.63	16.17
SD	±565.42	±943.47	±212.29	±70.10	±390.49	±46.28
Out-of-pocket payment	65.41	102.74	185.38	19	5.51	7.68
SD	±154.80	±238.25	±141.45	±39.90	±21.28	±16.08
**Total cost**	**4,584.9**	**10,493.19**	**6,954.93**	**311.48**	**1,287.91**	**1,012.95**
SD	±8,589.87	±13,324.10	±5,818.87	±547.47	±1,121.57	±1,585.77
Total cost without medication	4,412.51	10,194.8	6,924.09	271.92	975.28	996.78
SD	±8,585.93	±13,381.99	±5,521.75	±540.89	±922.67	±1,590.30

Inpatient care as a share of total expenditure remained substantial in both Tanzania (40.40%) and India (42.22%), but primary care had the greatest share of costs in India (50.72%). Tanzanian respondents included the highest expenditures for the justice system (6.21%); this could reflect a lack of access to healthcare services. Israel spent the lowest proportion (15.40%) of resources on inpatient care, while remaining expenditures were distributed relatively equally between community (35.12%) and primary care (33.01%), with a lesser share for outpatient treatment (13.35%).

## Relationships of factors with total costs

Results of country-specific GLM regression models in Table 4 show that gender was only significant in Tanzania where being a woman was associated with lower expenditure (*e*^b^ = 0.215; *p* = 0.000). Diagnosis was only significantly associated with total mental health costs in India, where compared to schizophrenia, a diagnosis of depression was associated with lower expenditure (*e*^b^ = 0.363; *p* = 0.017). Age and HoNOS scores (social disability) were not significantly associated with total costs in any country. The *R*^2^ of 0.16 indicates that 16% of the total cost variance could be explained by the variables in the regression model for Uganda, while the variance explanation for the other countries ranged between 4% and 7%. The residual plots in Supplementary eTables 1–5 indicate deviances from the requirement of normal distribution, particularly for the Tanzanian and for the Indian sample. While the Bayesian information criterion (BIC) suggests the best fit for the German model, the Akaike information criterion is lowest for the Tanzanian model. This indicates that the ratio between the sample size and the number of model variables is better in the German model.

**Table 4.** Generalized linear models (GLMs)^a^ for total healthcare expenditure for a 6-month period by country

	Germany		Uganda		Tanzania		India		Israel	
	*e*^b^	*p*	*e*^b^	*p*	*e*^b^	*p*	*e*^b^	*p*	*e*^b^	*p*
Women	0.860	0.534	0.950	0.697	0.215	0.000	0.714	0.301	1.344	0.097
Age (in years)	0.990	0.060	0.991	0.264	1.021	0.321	1.010	0.676	1.001	0.858
HoNOS total	1.022	0.143	1.023	0.050	1.010	0.667	0.990	0.509	0.982	0.185
Diagnosis ICD-10										
Schizophrenia	Reference category									
Depression	0.830	0.529	0.843	0.238	1.068	0.557	0.363	0.017	0.854	0.413
Other	0.802	0.490	_	_	_	_	0.252	0.061	0.831	0.538
*N*	170		137		102		93		103	
*R*^2^	0.04		0.05		0.16		0.07		0.04	
AIC	20.53635		16.40416		13.18043		15.78275		19.78349	
BIC	−588.8245		−554.1109		−265.9938		−231.9583		−392.9886	

AIC = Akaike information criterion; BIC = Bayesian information criterion.

a GLM with logit link function and gamma family error distribution.

The results of the GLM regression on total costs across all countries in Table 5 indicate that compared to Germany, mean expenditure per participant was significantly lower in Uganda (90%), Tanzania (99%) and India (86%). Given the significant interaction effects between female gender and country, female gender in Tanzania was associated with a 75% reduction in the total mental health service costs, relative to the costs for women in Germany. Additionally, the significant coefficient for the age × country interaction indicates Tanzanian healthcare costs increased by 3.6% for every additional year of age. There were no other country interaction effects with age, HoNOS or diagnosis. The *R*^2^ of 0.22 indicates that the overall model explains 22% of the variance in the total COI. The positive AIC and the negative BIC indicate a good ratio between the sample size and the number of model variables. The distribution of the residuals in Supplementary eFigure 6 indicates no deviance from the normal distribution.

**Table 5.** Generalized linear models for total healthcare expenditure in all five countries

		*e*^b^	*p*
Female		0.860	0.523
Country:			
• Germany		Reference	
• Uganda		0.105	0.001
• Tanzania		0.015	0.000
• India		0.138	0.040
• Israel		0.504	0.424
Female × country interaction:			
• Women × Germany		Reference	
• Women × Uganda		1.101	0.765
• Women × Tanzania		0.250	0.000
• Women × India		0.831	0.608
• Women × Israel		1.564	0.204
Age		0.985	0.053
Age × country interaction:			
• Age × Germany		Reference	
• Age × Uganda		1.007	0.623
• Age × Tanzania		1.036	0.039
• Age × India		1.023	0.211
• Age × Israel		1.016	0.258
HoNOS		1.022	0.133
HoNOS × country interaction:			
• HoNOS × Germany		Reference	
• HoNOS × Uganda		1.001	0.960
• HoNOS × Tanzania		0.989	0.640
• HoNOS × India		0.966	0.109
• HoNOS × Israel		0.961	0.103
Diagnosis			
• Schizophrenia		Reference	
• Depression		0.830	0.518
• Other		0.802	0.478
Diagnosis × country interaction:			
• Schizophrenia × Germany		Reference	
• Depression × Uganda		1.015	0.966
• Depression × Tanzania		1.290	0.521
• Depression × India		0.438	0.071
• Depression × Israel		1.028	0.944
• Other × Uganda		–	–
• Other × Tanzania		–	–
• Other × India		0.315	0.095
• Other × Israel		1.037	0.946
*N*	605		
*R*^2^	0.22		
AIC	17.50157		
BIC	−2,945.457		

AIC = Akaike information criterion; BIC = Bayesian information criterion.

## Discussion

While national income is correlated with the total COI in people living with mental disorders, it is a poor predictor of the sector-specific distribution of these expenditures and it can be argued that in these five settings, with vastly different systems, there is a need for more focus on primary care. Mean expenditure per study participant in Germany, one of the world’s wealthiest countries, is more than 33 times the mean expenditure in Tanzania, even when expressed in PPP-adjusted international dollars. That said, with the exception of Germany where 13.1% of the total health expenditure is on mental health, all countries spend 4% or less on these services. These expenditures are low relative to the burden of mental disorders.

Caution must be exercised in making comparisons on the balance of expenditure on care across these countries. Although at first glance Germany and Uganda have similar high proportions of expenditure on inpatient care in our study sample, the relative balance in expenditure on other services was very different. There is an almost complete reliance on inpatient services in Uganda (Kigozi *et al.*, 2016), with the highest mean number of inpatient stay nights of the five countries. While this analysis does not look specifically at the quality of inpatient care, other studies have previously raised fundamental issues about the protection of human rights and quality of inpatient care in Uganda (Molodynski *et al.*, 2017). While more recent evaluation suggests the situation in Uganda is improving (Atewologun *et al.*, 2025), it is critical to further assess the quality of inpatient care. Studies note the absence of mental health services in community settings, although there have been initiatives in rural areas, as part of the national mental health policy framework, to improve access to mental health services in communities by training nurses and primary care workers. However, even this reform did not allow nurses to provide consultations or prescribe medications; this was reported as a barrier to identification and timely treatment at clinics (Kigozi *et al.*, 2016). While there has been continued development of mental health within primary healthcare services in India (Gajera *et al.*, 2023), this does not appear to the case in Tanzania, where access to such care nationwide remains very limited (Anonymous, 2021).

Looking at differences in expenditure by participant characteristics, in contrast to previous international studies (Christensen *et al.*, 2020; König *et al.*, 2022; Ride *et al.*, 2020), we found no associations between healthcare costs and psychiatric diagnoses in any country except India. Similarly, the severity of functional impairment due to mental disorders was not associated with healthcare costs across all countries. Our findings are consistent with the results of a study in the UK (Twomey *et al.*, 2016) using the HoNOS as a measure for social disability. In contrast, results from other countries indicate clear positive associations between costs and social disability (Eagar *et al.*, 2004; König *et al.*, 2022).

One unexpected result of our study was the high cost for outpatient medications in Uganda, which could not be explained simply by the use of UK drug tariffs. However, this result corresponds with an earlier study revealing exceptionally high rates of prescription of second-generation antipsychotics in mental healthcare facilities in Uganda (Rukat, 2015; Rukat *et al.*, 2014). Furthermore, we learned from local study workers that the majority of mental health service users commonly received medications at the hospital pharmacy for free, after they were discharged from inpatient care. Unfortunately, we had no further information about the maintenance of drug use after medications received from hospital were used up. More generally, out-of-pocket payments for healthcare are a major barrier to service use in many low- and middle-income countries (Kazibwe *et al.*, 2021).

Another interesting finding was the low HoNOS (better) score in the Ugandan sample, significantly below the value expected in people with severe mental illness (Mirza *et al.*, 2021). These lower HoNOS values in the Uganda sample correspond with those from studies assessing the HoNOS at hospital discharge (Jansen *et al.*, 2019; Luo *et al.*, 2016), indicating a four-point reduction in HoNOS scores between hospital admission and discharge. This might perhaps be an indicator of improvements in the quality of care, but this needs to be assessed.

Even in high-income countries, there remain stark differences in the balance of services between inpatient care and community-based care. As Table 1 indicates, Germany has a very high rate of psychiatric beds, one of the highest in Europe, helping explain why expenditure is concentrated in psychiatric hospitals. Historically, the structure of the German healthcare system has put much less emphasis on primary care; the shift towards more community-based services is happening more gradually (Mueller-Stierlin *et al.*, 2022).

Israel has a much more balanced care system, relying more on primary and community care services; nonetheless, it still has extensive access to inpatient beds. Other studies also confirm Israel has a more well-balanced care system with less than 20% of expenditure on inpatient care and a very high proportion of community-based care (Roe *et al.*, 2021). Community services include many mobile treatments and services to support recovery: mobile multi-disciplinary (occupational therapist, social workers, psychologist, professional team director, etc.) mental health teams, specialist community team/support, vocational rehabilitation, sheltered workshops, vocational support centres and supported employment services (Gal *et al.*, 2021; Roe *et al.*, 2021). This more balanced care system is likely to have arisen in part from the implementation of an extensive mental health reform programme that was specifically focused on shifting care out of hospitals.

### Strengths and limitations

Our study is the first to investigate costs and factors associated with mental healthcare costs for people with severe mental illness in high-, middle- and low-income countries using individual patient-level data. This allows direct comparisons, for the first time, to be made on associations between service costs and social disability/diagnosis across the five countries.

Limitations result from small country-specific sample sizes and different approaches to study participants’ recruitment in each country. This makes it impossible to assess how representative service user samples are to the population of people with severe mental illness in study countries. The fit parameters of the country-specific GLM reveal differences depending on the different sample sizes. However, the overall model, including data from all countries, seems to fit the data best.

Furthermore, the results regarding the setting-specific distribution of costs must be considered with caution, because they might be biased by national selection procedures. We had to rely on the British National Health Service Drug Tariff for medication unit costs; this actually may be conservative, as the cost of generic medications varies considerably, relative to income in low- and lower-middle-income countries (Liu *et al.*, 2017). Moreover, these costs are typically funded out of pocket in low- and middle-income countries. A recent review of South Asian countries, including India, highlighted the risk of catastrophic healthcare costs associated with medications for many mental disorders (McDaid *et al.*, 2024). We also did not look at physical healthcare outcomes and expenditure, despite increased risk of co-morbidity (Hochman *et al.*, 2021; Liu *et al.*, 2017). Going forward, there is also scope, using the approach we have adopted, to make further comparisons between greater numbers of countries with different levels of World Bank income classification.

## Conclusions

Further research is needed to investigate the associations between the costs of mental healthcare, the balance between in- and outpatient treatment and the quality of mental healthcare in an international comparison. Differences in structure between inpatient treatment and community-based mental healthcare can be explored in future analyses to determine how they impact costs in relation to country-specific levels of economic development. There is also an opportunity to look at the management of mild and moderate mental disorders as services further develop. It would be of particular interest to assess the benefits of improved balance of care to mitigate against low levels of resources in low- and low-middle-income settings.

## Supporting information

10.1017/S2045796025100140.sm001 Park et al. supplementary material 1 Park et al. supplementary material

10.1017/S2045796025100140.sm002 Park et al. supplementary material 2 Park et al. supplementary material

10.1017/S2045796025100140.sm003 Park et al. supplementary material 3 Park et al. supplementary material

## Data Availability

The data that support the findings of this study will be available in the repository OPARU at https://oparu.uni-ulm.de/xmlui/ following an embargo until 31 December 2025 to allow for prioritized generation of research findings by members of the UPSIDES consortium (trial registration: ISRCTN, ISRCTN26008944, registered on 30 October 2019).

## Supplementary material

The supplementary material for this article can be found at https://doi.org/10.1017/S2045796025100140.
