# A comparison of the costs and patterns of expenditure for care for severe mental illness in five countries with different levels of economic development - CORRIGENDUM

**DOI:** 10.1017/S204579602510019X

**Published:** 2025-08-15

**Authors:** A-La Park, Oliver Jez, Reinhold Kilian, Ashleigh Charles, Jasmine Kalha, Palak Korde, Max Lachmann, Candelaria Mahlke, Galia Moran, Juliet Nakku, Fileuka Ngakongwa, Jackline Niwemuhwezi, Rebecca Nixdorf, Grace Ryan, Donat Shamba, Mike Slade, Tamara Waldmann

Author names Fileuka Ngakongwa and Tamara Waldmann updated following publication.
