# Supplementary material for: A comparison of the costs and patterns of expenditure for care for severe mental illness in five countries with different levels of economic development
Source: Epidemiol Psychiatr Sci. 2025 Jul 16;34:e40. doi: 10.1017/S2045796025100140 (PMC12281046; doi:10.1017/S2045796025100140)
Supplement: Park et al. supplementary material 1 — Park et al. supplementary material [file S2045796025100140sup001.docx]

# CHEERS 2022 Checklist

| **Topic** | **No.** | **Item** | **Location where item is reported** |
| --- | --- | --- | --- |
| **Title** |  |  |  |
|  | 1 | Identify the study as an economic evaluation and specify the interventions being compared. | Not reported |
| **Abstract** |  |  |  |
|  | 2 | Provide a structured summary that highlights context, key methods, results, and alternative analyses. | Abstract, Page 3, Lines 2-24 |
| **Introduction** |  |  |  |
| **Background and objectives** | 3 | Give the context for the study, the study question, and its practical relevance for decision making in policy or practice. | Introduction, Page 2- Lines 2 -24, Page 3 Lines 1 – 26, Page 4 Line 1 - 10. |
| **Methods** |  |  |  |
| **Health economic analysis plan** | 4 | Indicate whether a health economic analysis plan was developed and where available. | Methods, Page 4, Lines 11-24, Page 5 Lines 1 - 2 |
| **Study population** | 5 | Describe characteristics of the study population (such as age range, demographics, socioeconomic, or clinical characteristics). | Study sample Page 4, Lines 23-24, Page 5 Lines 3 -10 and Table 2. |
| **Setting and location** | 6 | Provide relevant contextual information that may influence findings. | Page 3, Lines 17-26, Page 4 Lines 1 – 5, Supplement |
| **Comparators** | 7 | Describe the interventions or strategies being compared and why chosen. | Not applicable. |
| **Perspective** | 8 | State the perspective(s) adopted by the study and why chosen. | Page 5, Line 19-25, page 6, Lines 1-3. |
| **Time horizon** | 9 | State the time horizon for the study and why appropriate. | Page 6, Lines 15-16 |
| **Discount rate** | 10 | Report the discount rate(s) and reason chosen. | Not applicable. |
| **Selection of outcomes** | 11 | Describe what outcomes were used as the measure(s) of benefit(s) and harm(s). | Page 7, Lines 2-7 |
| **Measurement of outcomes** | 12 | Describe how outcomes used to capture benefit(s) and harm(s) were measured. | Page 7, Lines 2-6. |
| **Valuation of outcomes** | 13 | Describe the population and methods used to measure and value outcomes. | Supplement |
| **Measurement and valuation of resources and costs** | 14 | Describe how costs were valued. | Page 6, Lines 14-19, |
| **Currency, price date, and conversion** | 15 | Report the dates of the estimated resource quantities and unit costs, plus the currency and year of conversion. | Page 6, Lines 14-19,  eTables 1-5. |
| **Rationale and description of model** | 16 | If modelling is used, describe in detail and why used. Report if the model is publicly available and where it can be accessed. | Not applicable. |
| **Analytics and assumptions** | 17 | Describe any methods for analysing or statistically transforming data, any extrapolation methods, and approaches for validating any model used. | Page 8, Lines 21-25.  Page 9 Lines 1-2. |
| **Characterising heterogeneity** | 18 | Describe any methods used for estimating how the results of the study vary for subgroups. | Page 6, Lines 21- 27, Page 7, Lines 1 - 7. |
| **Characterising distributional effects** | 19 | Describe how impacts are distributed across different individuals or adjustments made to reflect priority populations. | Page 6, Lines 21- 27, Page 7, Lines 1 - 7 |
| **Characterising uncertainty** | 20 | Describe methods to characterise any sources of uncertainty in the analysis. | Page 6, Lines 21- 27, Page 7, Lines 1 - 7. |
| **Approach to engagement with patients and others affected by the study** | 21 | Describe any approaches to engage patients or service recipients, the general public, communities, or stakeholders (such as clinicians or payers) in the design of the study. | Page 5, Lines 15-18. |
| **Results** |  |  |  |
| **Study parameters** | 22 | Report all analytic inputs (such as values, ranges, references) including uncertainty or distributional assumptions. | eTables 1-5. |
| **Summary of main results** | 23 | Report the mean values for the main categories of costs and outcomes of interest and summarise them in the most appropriate overall measure. | Page 8, Lines 15-25, Page 9, Lines 1-6, Table 3-5. |
| **Effect of uncertainty** | 24 | Describe how uncertainty about analytic judgments, inputs, or projections affect findings. Report the effect of choice of discount rate and time horizon, if applicable. | Page 9, Lines 8 – 25, Page 10 Lines 1-5.Figure 1, Table 1. |
| **Effect of engagement with patients and others affected by the study** | 25 | Report on any difference patient/service recipient, general public, community, or stakeholder involvement made to the approach or findings of the study | Not reported. |
| **Discussion** |  |  |  |
| **Study findings, limitations, generalisability, and current knowledge** | 26 | Report key findings, limitations, ethical or equity considerations not captured, and how these could affect patients, policy, or practice. | Discussion section, Pages 10-13. |
| **Other relevant information** |  |  |  |
| **Source of funding** | 27 | Describe how the study was funded and any role of the funder in the identification, design, conduct, and reporting of the analysis | Page 14, Lines 13-15 |
| **Conflicts of interest** | 28 | Report authors conflicts of interest according to journal or International Committee of Medical Journal Editors requirements. | Page 14, Lines 17-19. |
