# Supplementary material for: A comparison of the costs and patterns of expenditure for care for severe mental illness in five countries with different levels of economic development
Source: Epidemiol Psychiatr Sci. 2025 Jul 16;34:e40. doi: 10.1017/S2045796025100140 (PMC12281046; doi:10.1017/S2045796025100140)
Supplement: Park et al. supplementary material 2 — Park et al. supplementary material [file S2045796025100140sup002.docx]

**Supplementary Figures: eFigures 1 to 6**

**eFigure 1:GLM residual plots for the German sample**

**eFigure 2: GLM residual plot for the Ugandan sample**

**eFigure 3: GLM residual plot for the Tanzanian sample**

**eFigure 4: GLM residual plot for the Indian sample**

**eFigure 5: GLM residual plots for the Israeli sample**

**eFigure 6: GLM residual plots for the overall sample including participants from five countries**
