# Supplementary material for: A comparison of the costs and patterns of expenditure for care for severe mental illness in five countries with different levels of economic development
Source: Epidemiol Psychiatr Sci. 2025 Jul 16;34:e40. doi: 10.1017/S2045796025100140 (PMC12281046; doi:10.1017/S2045796025100140)
Supplement: Park et al. supplementary material 3 — Park et al. supplementary material [file S2045796025100140sup003.docx]

**Supplement 1: Unit costs in the five countries**

**eTable 1. Unit costs in Germany**

| **Inpatient services** | **Units** | **Unit costs (€)** | **Int$** |
| --- | --- | --- | --- |
| Acute psychiatric ward^1^ | Per night | 276.55 | 368.73 |
| Psychiatric rehabilitation ward^1^ | Per night | 276.55 | 368.73 |
| Long stay ward^1^ | Per night | 276.55 | 368.73 |
| General medical ward (physical health)^2,3^ | Per night | 649.91 | 866.55 |
| Addiction care services^1^ | Per night | 276.55 | 368.73 |
| Intensive care unit^2,3^ | Per night | 896.39 | 1195.19 |
| **Partial inpatient services** |  |  |  |
| Psychiatric outpatient clinic^4^ | Per visit | 201.16 | 268.21 |
| Psychiatric Emergency department^4^ | Per visit | 201.16 | 268.21 |
| General outpatient hospital (incl. emergency department)^3,4^ | Per visit | 201.16 | 268.21 |
| Psychiatric day hospital^3,4^ | Per visit | 201.16 | 268.21 |
| **Psychosocial services** | Per visit |  |  |
| Community mental health centre^1^ | Per visit | 201.16 | 268.21 |
| Day-care centre^1^ | Per visit | 201.16 | 268.21 |
| Group therapy^5^ | Per visit | 48.32 | 64.43 |
| Sheltered workshop (for people with disabilities)^6^ | Per visit | 47.26 | 63.01 |
| Specialist education^6^ | Per visit | 47.26 | 63.01 |
| **Outpatient services** |  |  |  |
| Psychiatrist^5^ | Per 1 hour | 128.16 | 170.88 |
| Psychologist^5^ | Per 1 hour | 102.78 | 137.04 |
| Primary care physician (or assistant)^5^ | Per visit | 12.68 | 16.91 |
| Other physician(s)^5^ | Per visit | 21.36 | 28.48 |
| Case manager^7^ | Per visit | 38.5 | 51.33 |
| Social worker^7^ | Per visit | 38.5 | 51.33 |
| Occupational therapist^8^ | Per visit | 68.20 | 90.93 |
| Home help / care worker^5^ | Per visit | 18.47 | 24.63 |
| Counsellor^9^ | Per visit | 43.90 | 58.53 |
| Career expert^10^ | Per visit | 39.72 | 52.96 |
| Traditional healer^11^ | Per visit | 16.4 | 21.87 |
| Religious practitioner^9^ | Per visit | 43.90 | 58.53 |
| **Criminal justice system** |  |  |  |
| Night at the police cell^12^ | Per night | 60.00 | 80 |
| Psychiatric assessment^13^ | Per investigation | 90.00 | 120 |
| **Other services** |  |  |  |
| Emergency department^1^ | Per visit | 201.16 | 268.21 |
| Psychosomatics^1^ | Per night | 276.55 | 368.73 |
| Geriatric psychiatry^1^ | Per night | 276.55 | 368.73 |
| Rehabilitation^14^ | Per night | 100.94 | 134.59 |
| Daycare^15^ | Per visit | 190.00 | 253.33 |
| Home treatment^1^ | Per visit | 276.55 | 368.73 |
| Cure^14^ | Per night | 100.94 | 134.59 |
| Outpatient social psychiatry^9^ | per visit | 43.90 | 58.53 |
| Integrated care^14^ | per visit | 39.00 | 52 |
| Physical therapy^5^ | per visit | 21.36 | 28.48 |
| Osteopathy^5^ | per visit | 21.36 | 28.48 |
| Speech therapy^16^ | per visit | 60.24 | 80.32 |
| Riding therapy^14^ | per visit | 12.71 | 16.95 |
| Integration specialist service^9^ | per visit | 43.90 | 58.53 |
| Sociotherapy^9^ | per visit | 43.90 | 58.53 |
| Assisted living (supported housing)^6^ | per visit | 47.26 | 63.01 |
| Addiction counselling^9^ | per visit | 43.90 | 58.53 |
| Dance therapy^14^ | per visit | 12.71 | 16.95 |
| Couple therapy^5^ | per visit | 102.78 | 137.04 |
| Youth welfare office^7^ | per visit | 38.5 | 51.33 |
| Physiotherapy^7^ | per visit | 21.36 | 28.48 |
| Nursing station^14^ | per visit | 17.00 | 22.67 |
| Legal guardian^17^ | Per night | 6.66 | 8.88 |
| Education companion^9^ | Per visit | 43.90 | 58.53 |
| Health department expert evidence^18^ | Per visit | 100.00 | 133.33 |
| Probation officer^7^ | Per visit | 38.5 | 51.33 |

**eTable 2. Unit costs in Israel**

| **Inpatient services** | **Units** | **Unit costs (ILS)** | **Int$** |
| --- | --- | --- | --- |
| Acute psychiatric ward^19^ | Per night | 1309 | 358.63 |
| Psychiatric rehabilitation ward^19^ | Per night | 1468 | 402.19 |
| Long stay ward^19^ | Per night | 1008 | 276.16 |
| Emergency/crisis centre^19^ | Per night | 980 | 268.49 |
| General medical ward (physical health)^19^ | Per night | 1309 | 358.63 |
| Psychotrauma services^19^ | Per night | 135 | 36.99 |
| Occupational therapy^19^ | Per night | 158 | 43.29 |
| **Partial inpatient services** |  |  |  |
| Psychiatric outpatient clinic^19^ | Per visit | 495 | 135.62 |
| Psychiatric Emergency department^19^ | Per visit | 980 | 268.49 |
| General outpatient hospital (incl. emergency department)^19^ | Per visit | 737.5 | 202.05 |
| Psychiatric day hospital^19^ | Per visit | 490 | 134.25 |
| **Psychosocial services** |  |  |  |
| Community mental health centre^19^ | Per visit | 495 | 135.62 |
| Day-care centre^19^ | Per visit | 168 | 46.02 |
| Group therapy^19^ | Per visit | 135 | 36.99 |
| Sheltered workshop (for disabled)^19^ | Per night | 42.06 | 11.53 |
| Community mental health services by NGOs^19^ | Per visit | 495 | 135.62 |
| **Outpatient services** |  |  |  |
| Psychiatrist^19^ | Per visit | 495 | 135.62 |
| Psychologist^19^ | Per visit | 290 | 79.45 |
| Primary care physician (or assistant)^19^ | Per visit | 495 | 135.62 |
| Other physician(s)^19^ | Per visit | 495 | 135.62 |
| District nurse / Community psychiatric nurse^19^ | Per visit | 133 | 36.44 |
| Advanced nurse practitioners / Practise nurse^19^ | Per visit | 133 | 36.44 |
| Case manager^20^ | Per visit | 56 | 15.34 |
| Social worker^20^ | Per visit | 56 | 15.34 |
| Occupational therapist^21^ | Per visit | 92 | 25.21 |
| Home help / care worker^20^ | Per visit | 56 | 15.34 |
| (Non-mental health) Services by NGOs^20^ | Per visit | 56 | 15.34 |
| Peer support worker (PSW) ^20^ | Per visit | 56 | 15.34 |
| Counsellor^20^ | Per visit | 56 | 15.34 |
| Career expert^20^ | Per visit | 56 | 15.34 |
| Religious practitioner^22^ | Per visit | 63 | 17.26 |
| **Criminal justice system** |  |  |  |
| Psychiatric assessment^19^ | Per investigation | 756 | 207.12 |
| **Other services** |  |  |  |
| Clubhouse^20^ | Per visit | 56 | 15.34 |
| Social club^20^ | Per visit | 135 | 36.99 |
| Comprehensive hostel^20^ | Per visit | 1468 | 402.19 |
| Rehabilitation mentor^19^ | Per visit | 35 | 9.59 |
| Employment coordinator^20^ | Per visit | 56 | 15.34 |
| Social work student^20^ | Per visit | 56 | 15.34 |
| Body-soul therapist^20^ | Per visit | 92 | 25.21 |
| Mentoring service ^20^ | Per visit | 56 | 15.34 |
| Business facilitator^20^ | Per visit | 56 | 15.34 |

**eTable 3. Unit costs in Tanzania**

| **Inpatient services** | **Units** | **Unit costs (TZS)** | **Int$** |
| --- | --- | --- | --- |
| Acute psychiatric ward^23^ | per night | 66 428.5 | 72.88 |
| Psychiatric rehabilitation ward^23^ | per night | 66 428.5 | 72.88 |
| Long stay ward^23^ | per night | 66 428.5 | 72.88 |
| Emergency/crisis centre^23^ | per night | 66 428.5 | 72.88 |
| General medical ward (physical health)^23^ | per night | 51 000 | 55.95 |
| Recovery college^23^ | per night | 6 666.7 | 7.31 |
| Psychotrauma services^23^ | per night | 66 428.5 | 72.88 |
| Occupational therapy^23^ | per night | 31 666.67 | 34.74 |
| Electroconvulsive therapy^23^ |  | 20 833.35 | 22.86 |
| **Partial inpatient services** |  |  |  |
| Psychiatric outpatient clinic^23^ | per visit | 31 666.67 | 34.74 |
| Psychiatric Emergency department^23^ | per visit | 31 666.67 | 34.74 |
| General outpatient hospital (incl. emergency department)^23^ | per visit | 10 000 | 10.97 |
| Psychiatric day hospital^23^ | per visit | 31 666.67 | 34.74 |
| Trauma centre^23^ | per visit | 31 666.67 | 34.74 |
| Policlinic of NGO (for physical & mental health)^23^ | per visit | 30 000 | 32.91 |
| **Psychosocial services** |  |  |  |
| Community mental health centre^23^ | per visit | 31 666.67 | 34.74 |
| Group therapy^23^ | per visit | 15 000 | 16.46 |
| Specialist education^23^ | per visit | 16 666.67 | 18.28 |
| **Outpatient services** |  |  |  |
| Psychiatrist^23^ | per visit | 40 000 | 43.88 |
| Psychologist^23^ | per visit | 40 000 | 43.88 |
| Primary care physician (or assistant)^23^ | per visit | 40 000 | 43.88 |
| Other physician(s)^23^ | per visit | 40 000 | 43.88 |
| District nurse / Community psychiatric nurse^23^ | per visit | 16 666.67 | 18.28 |
| Advanced nurse practitioners / Practise nurse^23^ | per visit | 16 666.67 | 18.28 |
| Case manager^23^ | per visit | 16 666.67 | 18.28 |
| Social worker^23^ | per visit | 16 666.67 | 18.28 |
| Occupational therapist^23^ | per visit | 16 666.67 | 18.28 |
| Home help / care worker^23^ | per visit | 16 666.67 | 18.28 |
| Peer support worker (PSW)^23^ | per visit | 16 666.67 | 18.28 |
| Counsellor^23^ | per visit | 35 000 | 38.4 |
| Traditional healer^23^ | per visit | 20 000 | 21.94 |
| Religious practitioner^23^ | per visit | 2 000 | 2.19 |
| **Criminal justice system** |  |  |  |
| Night at the police cell^23^ | per night | 51 000 | 55.95 |
| **Other services** |  |  |  |
| Rehabilitation college/facility^23^ | per visit | 66 428.5 | 72.88 |

**eTable 4. Unit costs in Uganda**

| **Inpatient services** | **Units** | **Unit costs (UGX)** | **Int$** |
| --- | --- | --- | --- |
| Acute psychiatric ward^24^ | per night | 50 000 | 35.97 |
| Psychiatric rehabilitation ward^24^ | per night | 45 000 | 32.37 |
| Long stay ward^24^ | per night | 25 000 | 17.99 |
| Emergency/crisis centre^24^ | per night | 50 000 | 35.97 |
| General medical ward (physical health)^24^ | per night | 40 000 | 28.78 |
| Recovery college^24^ | per night | 20 000 | 14.39 |
| Addiction care services^24^ | per night | 30 000 | 21.58 |
| Psychotrauma services^24^ | per night | 20 000 | 14.39 |
| Occupational therapy^24^ | per night | 34 000 | 24.46 |
| **Partial inpatient services** |  |  |  |
| Psychiatric outpatient clinic^24^ | per visit | 10 000 | 7.19 |
| Psychiatric Emergency department^24^ | per visit | 10 000 | 7.19 |
| General outpatient hospital (incl. emergency department)^24^ | per visit | 10 000 | 7.19 |
| Trauma centre^24^ | per visit | 20 000 | 14.39 |
| Policlinic of NGO (for physical & mental health)^24^ | per visit |  |  |
| Community recovery centre^24^ | per visit | 20 000 | 14.39 |
| **Psychosocial services** |  |  |  |
| Community mental health centre^24^ | per visit | 20 000 | 14.39 |
| Day-care centre^24^ | per visit | 34 000 | 24.46 |
| Group therapy^24^ | per visit | 5 000 | 3.6 |
| Recovery College^24^ | per visit | 20 000 | 14.39 |
| **Outpatient services** |  |  |  |
| Psychiatrist^24^ | per visit | 40 000 | 28.78 |
| Psychologist^24^ | per visit | 20 000 | 14.39 |
| Primary care physician (or assistant)^24^ | per visit | 30 000 | 21.58 |
| Other physician(s)^24^ | per visit | 30 000 | 21.58 |
| District nurse / Community psychiatric nurse^24^ | per visit | 15 000 | 10.79 |
| Advanced nurse practitioners / Practise nurse^24^ | per visit | 15 000 | 10.79 |
| Case manager^24^ | per visit | 20 000 | 14.39 |
| Social worker^24^ | per visit | 20 000 | 14.39 |
| Occupational therapist^24^ | per visit | 20 000 | 14.39 |
| Home help / care worker^24^ | per visit | 25 000 | 17.99 |
| (Non-mental health) Services by NGOs^24^ | per visit | 25 000 | 17.99 |
| Peer support worker (PSW)^24^ | per visit | 20 000 | 14.39 |
| Counsellor^24^ | per visit | 20 000 | 14.39 |
| Career expert^24^ | per visit | 25 000 | 17.99 |
| Traditional healer^24,25^ | per visit | 1 Ziege | 86.33 |
|  | per visit | =120 000UGX |  |
| Religious practitioner^24^ | per visit | 10% of income | 203.6 |
|  |  | = 283 000UGX |  |
| **Criminal justice system** |  |  |  |
| Night at the police cell^24^ | per night | 50 000 | 35.97 |
| Psychiatric assessment^24^ | per investigation | 40 000 | 28.78 |

**eTable 5. Unit costs in India**

| **Inpatient services** | **Units** | **Unit costs (2015USD/INR)** | **Int$** |
| --- | --- | --- | --- |
| Acute psychiatric ward^26^ | per night | 9.85/734.81 | 33.31 |
| Psychiatric rehabilitation ward^26^ | per night | 9.85/734.81 | 33.31 |
| Long stay ward^26^ | per night | 9.85/734.81 | 33.31 |
| Emergency/crisis centre^26^ | per night | 9.85/734.81 | 33.31 |
| General medical ward (physical health)^26^ | per night | 9.85/734.81 | 33.31 |
| Recovery college^26^ | per night | 9.85/734.81 | 33.31 |
| Addiction care services^26^ | per night | 9.85/734.81 | 33.31 |
| Psychotrauma services^26^ | per night | 9.85/734.81 | 33.31 |
| Occupational therapy^26^ | per night | 9.85/734.81 | 33.31 |
| **Partial inpatient services** |  |  |  |
| Psychiatric outpatient clinic^26^ | per visit | 2.79/208.13 | 9.43 |
| Psychiatric Emergency department^26^ | per visit | 2.79/208.13 | 9.43 |
| General outpatient hospital (incl. emergency department) | per visit | 2.79/208.13 | 9.43 |
| Psychiatric day hospital^26^ | per visit | 2.79/208.13 | 9.43 |
| Trauma centre^26^ | per visit | 2.79/208.13 | 9.43 |
| Policlinic of NGO (for physical & mental health)^26^ | per visit | 2.79/208.13 | 9.43 |
| Community recovery centre^26^ | per visit | 2.79/208.13 | 9.43 |
| **Psychosocial services** |  |  |  |
| Community mental health centre^26^ | per visit | 0.6/44.76 | 2.03 |
| Day-care centre^26^ | per visit | 0.6/44.76 | 2.03 |
| Sheltered workshop (for people living with disabilities)^27^ | per visit | 18.0/1342.8 | 60.9 |
| **Outpatient services** |  |  |  |
| Psychiatrist^26^ | per visit (60min) | 25.2/1879.92 | 85.22 |
| Psychologist^26^ | per visit (60min) | 3.0/223.8 | 10.15 |
| Primary care physician (or assistant)^26^ | per visit (60min) | 21.0/1566.6 | 71.02 |
| District nurse / Community psychiatric nurse^26^ | per visit (60min) | 6.0/447.6 | 20.3 |
| Advanced nurse practitioners / Practise nurse^26^ | per visit (60min) | 6.0/447.6 | 20.3 |
| Social worker | per visit (60min) | 6.0/447.6 | 20.3 |
| Peer support worker (PSW)^26^ | per visit | 6.0/447.6 | 20.3 |
| Counsellor^26^ | per visit (60min) | 6.0/447.6 | 20.3 |
| **Criminal justice system** |  |  |  |
| Night at the police cell^26^ | per nitght | 9.85/734.81 | 33.31 |

**References**

1. GKG. PEPP-Entgelttarif 2022 für Krankenhäuser im Anwendungsbereich der BPflV und Unterrichtung des Patienten gemäß § 8 Abs. 5 BPflV. In: Bamberg: Gemeinnützige Krankenhausgesellschaft des Landkreises Bamberg mbH; 2022: <https://gkg-bamberg.de/wp-content/uploads/2021/02/PEPP-Entgelttarif_compressed.pdf>.

2. InEK. Fallpauschalen-Katalog 2021 <https://www.g-drg.de/ag-drg-system-2021/fallpauschalen-katalog/fallpauschalen-katalog-2021>. Published 2020. Accessed 30.03, 2024.

3. VDEK. Landesbasifallwerte 2021. <https://www.vdek.com/vertragspartner/Krankenhaeuser/landesbasisfallwerte.html>. Published 2021. Accessed 03.05, 2023.

4. InEK. PEPP Entgeltkatalog 2021. <https://www.g-drg.de/PEPP-Entgeltsystem_2021/PEPP-Entgeltkatalog>. Published 2020. Accessed 11.11, 2023.

5. KBV. *Einheitlicher Bewertungsmaßstab (EBM).* Berlin2021.

6. Workshop operator Günzburg (Werkstättenbetreiber Günzburg). Peronal communication. Sheltered workshop costs. In:2018.

7. Kreiss Pinneberg. *Steckbrief Sozialpädagogische Familienhilfe (Profile Social-educational family support).* Elmshorn: Kreiss Pinneberg,;2018.

8. VDEK. *Preisliste Leistungen Krankengymnastik/Physiotherapie, Massagen und medizinische Bäder (Price list services physiotherapy/physiotherapy, massages and medical baths).* VDEK;2021.

9. Personal communication with Caritas employee. In:2021.

10. Federal Employment Agency. *2017 Annual Report by the Federal Employment Agency.* Nuremberg: Federal Employment Agency;2017.

11. Baden-Württemberg Municipal Supply Association (KVBW). *Fee schedule for alternative practitioners.* Stuttgart: KVBW;2017.

12. Tilmann Grewe. Polizeigewahrsam: So viel kostet eine Nacht in der Zelle. January 26, 2018.

13. Federal Ministry of Justice. Law on the remuneration of experts, interpreters, translators and the compensation of honorary judges, witnesses and third parties (Judicial Remuneration and Compensation Act - JVEG). In*.* Vol Judicial Remuneration and Compensation Act of May 5, 2004 (BGBl. I p. 718, 776), which was last amended by Article 17 of the law of June 25, 2021 (BGBl. I p. 2154)"2021.

14. Personal communication. In:2021.

15. GKV Spitzernverband. *Pauschalierende Entgelte für Psychiatrie und Psychosomatik (PEPP) gem. § 7 S. 1 Nr. 1 BPflV i.V.m. § 1 Abs. 1 PEPPV 2017* GKV Spitzernverband,;2017.

16. GKV Spitzernverband. *Übergangsänderungsvereinbarung zum Vertrag nach § 125 Abs. 1 SGB V über die Versorgung mit Leistungen der Stimm-, Sprech-, Sprach- und Schlucktherapie und deren Vergütung* GKV Spitzernverband,;2017.

17. Federal Ministry of Justice. Act on the remuneration of guardians and carers (Guardians and Carers Remuneration Act - VBVG). In*.* Vol Guardian and Carer Remuneration Act of May 4, 2021 (BGBl. I p. 882, 925), which was amended by Article 8 of the law of June 24, 2022 (BGBl. I p. 959)"2021.

18. Driemecker H. *Medical reports to clarify the facts of the case. Expert evidence - report costs.* 2021.

19. UPSIDES Team Israel. Personal communication. In:2021.

20. Salaryexpert.com. Average social worker gross salary in Israel. <https://www.salaryexpert.com/salary/job/social-worker/israel/sedom>. Published 2021. Accessed.

21. Salaryexpert.com. Average occupational therapist gross salary. <https://www.salaryexpert.com/salary/job/occupational-therapist/israel> Published 2021. Accessed.

22. Institute ER. Rabbi Salary in Israel. <https://www.erieri.com/salary/job/rabbi/israel#:~:text=The%20average%20pay%20for%20a,of%20education%20for%20a%20Rabbi.%20D49111>. Published 2021. Accessed.

23. UPSIDES Team Tanzania. Personal communication. In:2021.

24. UPSIDES Team Uganda. Personal communication. In:2021.

25. Salaryexpert.com. Average salary in Uganda. <https://www.salaryexplorer.com/average-salary-wage-comparison-uganda-c225>. Published 2021. Accessed.

26. Chisholm D, Garman E, Breuer E, et al. Health service costs and their association with functional impairment among adults receiving integrated mental health care in five low- and middle-income countries: the PRIME cohort study. *Health Policy Plan.* 2020;35(5):567-576.

27. Personal communication with 3 social workers. In:2021.
